# Green Synthesis of Fluorescent Palladium Nanoclusters

**DOI:** 10.3390/ma11020191

**Published:** 2018-01-26

**Authors:** Yan Peng, Pei Wang, Liang Luo, Lang Liu, Fu Wang

**Affiliations:** 1 College of Chemistry and Chemical Engineering, Xinjiang University, Urumqi 830046, Xinjiang, China; yanpeng9236@sina.com; 2 Laboratory of Environmental Sciences and Technology, Xinjiang Technical Institute of Physics & Chemistry, Chinese Academy of Sciences, Urumqi 830011, Xinjiang, China; 13126815560@163.com (P.W.); luoliang@ms.xjb.ac.cn (L.L.)

**Keywords:** palladium nanoclusters, methionine, fluorescence, hemoglobin

## Abstract

Metal nanoclusters, with dimensions between atomic and nanoparticles, have attracted a great deal of attention due to their significantly unusual properties. Water-soluble palladium nanoclusters (Pd NCs) with blue-green fluorescence were synthesized by a water bath heating method, with methionine as a stabilizer and ascorbic acid as a reducing agent. We investigated the optimal synthesis conditions, stability, and pH response of the obtained products in detail. The synthesized materials were characterized by ultraviolet-absorption spectroscopy, fluorescence spectroscopy, high-resolution transmission electron microscopy, and atomic force microscopy. These experimental results showed that the Pd NCs had a small size of ~1.91 nm, with a uniform size distribution. Additionally, the Pd NCs emitted blue-green fluorescence under ultraviolet light with a quantum yield of 5.47%. Notably, both stabilizers and reducing agents used in this synthesis method are nutrients for humans, non-toxic, and harmless. This method could be viewed as a biologically friendly and green way of preparing fluorescent metal nanoclusters. The as-prepared fluorescent Pd NCs also possessed excellent fluorescence detection ability and were very sensitive to low concentrations of hemoglobin, with a linear response in the range of 0.25–3.5 μM and a detection limit of 50 nM.

## 1. Introduction

Metal nanoclusters (NCs), are an important kind of fluorescent nanomaterial, which have recently received considerable attention [1,2]. NCs consisting of several to tens of metal atoms are often of a comparable size to the Fermi wavelength of conduction electrons, which results in molecule-like properties, including discrete electronic states and size-dependent fluorescence [3,4,5]. NCs have some properties similar to molecules but also feature characteristics of nanomaterials [6,7]. Unlike traditional fluorescent nanoprobes (such as organic dye molecules, fluorescent protein, nanoparticles, and semiconducting quantum dots), metal NCs exhibit unusual physical, optical, and electrical properties, such as strong luminescence and low toxicity, owing to their unique electronic structure and ultra-small size [8,9,10]. Many kinds of metal NCs have been synthesized by changing ligands or biological scaffolds, which have broad applications in the fields of biosensing, fluorescence imaging, catalysis, and single molecule optoelectronic devices [11,12,13,14].

To date, the rational design, controllable synthesis, and applications of ultra-small NCs have drawn considerable interest [15,16,17]. The synthesis of ultra-small NCs can be achieved through top-down and bottom-up synthetic routes. Through the use of the top-down method, smaller clusters are prepared by etching larger nanoparticles or larger bulk material [18]. In the bottom-up approach, the corresponding metal precursors are reduced to atoms with a reducing reagent and then the metal clusters are formed by nucleation of the zero-valence metal atoms [19]. Among these approaches, bottom-up methods have been widely used as a synthesis method because of the convenience of preparation and ease of control over the shape and size of the NCs. Brust et al. first used a two-phase liquid reduction method to synthesize stable small-size gold nanoclusters in 1994 [20]. Since then, through the use of modified Brust–Schiffrin methods (one-phase and two-phase), gold, silver, copper, and platinum nanoclusters of different core sizes have been successfully synthesized with different protecting ligands and reducing reagents. Zheng et al. prepared dendrimer-stabilized gold nanoclusters using poly(amidoamine) (PAMAM) as a stabilizer and NaBH_4_ to reduce HAuCl_4_, with a luminescence quantum yield exceeding 40% [21]. Huang et al. prepared fluorescent silver nanoclusters with DNA as a stabilizer, with a quantum yield of 66.2% [22]. Cao et al. used tannic acid as a stabilizer and reduced copper sulfate with ascorbic acid to produce blue-emitting copper clusters with a quantum yield of 14%, which is much higher than that of fluorescent copper clusters prepared by other researchers [23]. Tanaka et al. also used the same stabilizers and reducing agents to prepare luminescent platinum clusters with quantum efficiencies as high as 18% [24].

Research on fluorescent gold, silver, copper, and platinum metal nanoclusters has matured, and regulation of the emission spectrum and quantum efficiency can be achieved to a certain extent [25]. However, there have been few reports on the synthesis and properties of fluorescent palladium nanoclusters (Pd NCs). The possible reason for this lack of studies is that the main precursor, PdCl_2_ is not easily soluble in water, and reduction of palladium is a relatively slow process. Nano-sized Pd plays an important role in different types of chemical reactions and exhibits different characteristics from those of nanoparticles, such as gold and silver. Pd nanoparticles can catalyze C–C cross-couplings [26], olefin hydrogenation [27], and CO oxidation [28]. The properties of nanomaterials are often related to their size, hence various efforts have been made to control the formation of Pd nanoparticles [29,30,31]. Although considerable progress has been made, the preparation of small (<2 nm) Pd NCs with fluorescence remains challenging. Recent studies have shown that Pd NCs of very small size may be formed when the surface is effectively passivated. Obora’s group developed a one-pot strategy for the synthesis of Pd NCs in *N*,*N*-dimethylformamide (DMF) solution [32,33]. Although small Pd nanoclusters were successfully prepared with good size control, the synthesized materials were dissolved in organic solvents with the aid of capping agents. The use of these conditions makes it difficult to collect and purify these nanoclusters. Furthermore, DMF is a toxic organic reagent, affecting human eyes, skin, and the respiratory tract to varying degrees. Therefore, the design of a non-toxic and green method for the synthesis of water-soluble fluorescence Pd NCs would be highly desirable.

**Scheme 1:**
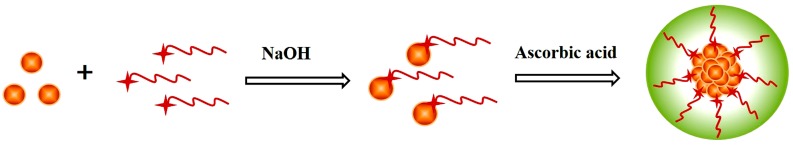
Schematics of the formation of fluorescence Pd NCs.

Inspired by this method, we synthesized Pd NCs using methionine as a stabilizer, ascorbic acid as reducing agent, and ammonium tetrachloropalladate(II) as metal precursor via water bath heating (Scheme 1). Bio-molecules (methionine) were used as a sealing agent and stabilizer due to their exhibited low toxicity and good biocompatibility. Ammonium tetrachloropalladate(II) was used as a precursor owing to its good solubility in water. Compared with sodium borohydride or hydrazine hydrate, ascorbic acid is a milder, environmentally-friendly reducing agent and the degree of the reaction could be easily controlled to achieve Pd NCs in the preparation process. The prepared Pd NCs could be well dispersed in water and had a size of ~1.91 nm. Under 365 nm UV lamp irradiation, the solution of Pd NCs emitted blue-green fluorescence, with a quantum yield of approximately 5.47%. We also found that the fluorescence of the nanoclusters was sensitively and selectively quenched by hemoglobin. Therefore, a hemoglobin sensing platform was established and studied based on our Pd NCs. Our prepared Pd NCs show potential for applications in bio-imaging and catalysis owing to their low toxicity and narrow size distribution.

## 2. Materials and Methods 

### 2.1. Chemicals and Reagents 

Ammonium tetrachloropalladate (II) ((NH_4_)_2_PdCl_4_, 98%) and dl-methionine (C_5_H_11_NO_2_S, 99.9%) were obtained from Adamas-Beta (Shanghai, China). Ascorbic acid (C_6_H_8_O_6_, 99.9%), human serum albumin (HSA), ribonuclease A (RNA), cytochrome C, transferrin, bovine serum albumin (BSA), lysozyme, and hemoglobin were obtained from Solarbio (Beijing, China). All competitive metal ions and glucose used for selectivity testing were acquired from Sinopharm Chemical Reagent Co. Ltd. (Shanghai, China). All reagents were used as received without further purification. Ultrapure water (resistivity: 18.2 MΩ⋅cm) was obtained from a Millipore purification system.

### 2.2. Instruments

UV–vis absorption spectra were recorded on a Shimadzu UV-1800 spectrophotometer (Kyoto, Japan). Fluorescence spectra were performed on a Hitachi F-7000 fluorescence spectrometer (Tokyo, Japan). High-resolution transmission electron microscopy (HR-TEM) data were obtained on a FEI Tecnai G2 F20 S-TWIN transmission electron microscopy instrument operating at 200 kV (Hillsboro, OR, USA). Time-resolved luminescence intensity decays were recorded on a Horiba JY Fluorolog-3 molecule fluorometer (Paris, France), and samples were excited by a 395-nm laser light source. Atomic force microscopy (AFM) data were obtained on a Bruker MultiMode8 (Leipzig, Germany).

### 2.3. Synthesis of Pd NCs

All glassware used in the following procedures was thoroughly cleaned with freshly prepared aqua regia (3:1 conc. HCl/HNO_3_ v/v) and rinsed with ultrapure water prior to use. In a typical procedure, 12 mL of (NH_4_)_2_PdCl_4_ aqueous solution (2.5 mM) was mixed with 24 mL of methionine solution (0.1 M), and 3.6 mL of NaOH (0.6 M) for 30 min, and then 9 mL of l-ascorbic acid (0.14 M) was added into the solution at 60 °C. Within 5.5 h, yellow solutions of Pd NCs were obtained. Afterwards, the reaction solution was centrifuged at 8000 rpm for 10 min to remove large particles and dialyzed with water via a dialysis membrane (1000 Da) to remove the free ions and ligand. The resulting solution was stored in the dark at 4 °C for use. 

### 2.4. Fluorescent Detection of Hemoglobin 

To evaluate the sensitivity toward hemoglobin, 100 µL of hemoglobin solution of various concentrations was added into 1.9 mL of the prepared Pd NCs solutions and the mixtures were incubated at room temperature before spectral measurements. To check the selectivity of the Pd NCs, a series of competitive molecules at 5 µM were tested, including HSA, RNA, cytochrome C, transferrin, BSA, lysozyme, glucose, K^+^, Ca^2+^, Na^+^, Mg^2+^, CO_3_^2−^, and Cl^−^ were respectively introduced to a group of Pd NCs solutions (1.9 mL) to measure the change of fluorescence intensity. The fluorescence spectra were recorded at room temperature with excitation at 420 nm; both the excitation and emission slit widths were 5 nm.

## 3. Results and Discussion

### 3.1. Synthesis of Pd NCs

**Figure 1:**
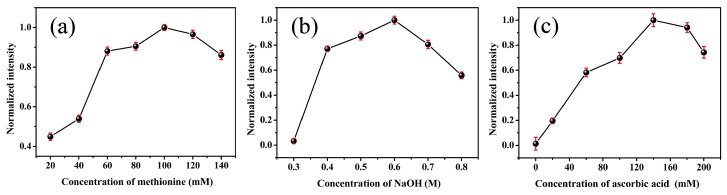

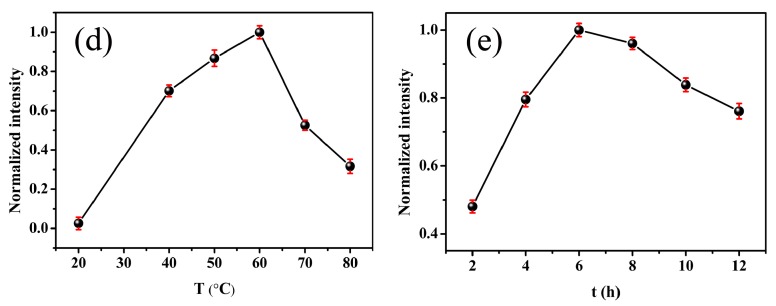
The influence of the concentration of methionine (**a**); the concentration of NaOH (**b**); the concentration of ascorbic acid (**c**); the reaction temperature (**d**); and time (**e**) on the fluorescence intensity of Pd NCs.

In the process of synthesizing Pd NCs, the aqueous solution of the metal precursor (NH_4_)_2_PdCl_4_ was first reacted with the ligand (methionine) in aqueous solution under alkaline conditions to form a low valence metal-stabilizer complex and then further reduced under the action of the reducing agent ascorbic acid to generate methionine-coated zero-valent palladium metal nanoclusters. These results showed that the amount of ligand methionine, the concentration of sodium hydroxide, the concentration of reductant ascorbic acid, and the reaction temperature and time affected the formation of Pd NCs. The reaction conditions of the Pd NCs synthesis were optimized. As shown in Figure 1a, the effects of the methionine concentration on the synthesis were first investigated under the same conditions. These results showed that the highest fluorescence of the Pd NCs was obtained when the concentration of methionine was 100 mM. This result might be related to the solubility of methionine. Alkaline conditions were beneficial for the generation of metal nanoclusters. Figure 1b shows that fluorescent nanoclusters could not be formed when the concentration of NaOH was low and that the fluorescence emission intensity of Pd NCs gradually increased as the concentration of NaOH was increased from 0.3 to 0.6 M. When the concentration was 0.6 M, the fluorescence emission intensity of the nanoclusters was optimal, but started to decrease for further increases in the concentration of NaOH. Harsh alkaline reaction conditions are typically necessary for preparing stable metal nanoclusters. The amount of reducing agent also directly affects the size of the nanocluster. An excessively strong reducing agent might directly generate large nanoparticles; conversely if the reagent is too weak, it may not be sufficient to initiate nucleation from the metal-stabilizer complex [34,35]. As shown in Figure 1c, when the concentration of the ascorbic acid reducing agent was 0.14 M, the best reduction effect was achieved, and the fluorescence intensity of the nanoclusters reached its highest value. The reaction time and reaction temperature are also important factors affecting the properties of nanoclusters. As shown in Figure 1d, as the reaction temperature was increased, the fluorescence emission intensity of the nanoclusters gradually increased. When the reaction temperature was 60 °C, the maximum fluorescence emission intensity was obtained. However, as the temperature was increased further, the fluorescence clearly decreased. Thus, excessively high or low reaction temperatures were not conducive to the formation of small size nanoclusters. We speculate that the temperature might affect the structure of methionine and that it shows the greatest stability at an optimum temperature. As Figure 1e shows, as the reaction time was extended, the fluorescence emission intensity of the Pd NCs gradually increased and reached a maximum at ~6 h. The reaction time could be extended by prolonging the reaction time. Therefore, we selected 6 h as the optimal reaction time. We selected optimized conditions to prepare Pd NCs as follows: methionine 100 mM; NaOH 0.6 M; ascorbic acid 0.14 M, temperature 60 °C; time 6 h. 

### 3.2. Characterization of Pd NCs

**Figure 2:**
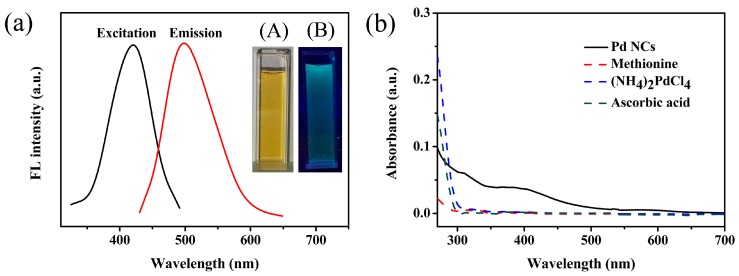
(**a**) Fluorescence excitation and emission spectra of the as-synthesized Pd NCs. Inset shows photographs of the Pd NCs aqueous solution in room light (A) and UV light (B); (**b**) UV-vis absorption spectra of the Pd NCs, methionine, (NH_4_)_2_PdCl_4_ and ascorbic acid.

Photoluminescence is a typical property of small-size palladium clusters because of the formation of discrete energy levels. As shown in the inset of Figure 2a, the Pd NCs appeared as a yellow solution under visible light (A) and emitted blue-green fluorescence under irradiation at 365 nm (B). The maximum excitation wavelength of the synthesized palladium clusters was 420 nm and the optimal emission wavelength was 500 nm (Figure 2a). The Stokes shift of the Pd NCs was calculated to be 80 nm. A Stokes shift of less than 150 nm can be attributed to interband transitions of electrons in the Pd NCs. Figure 2b shows UV–vis absorption spectra of the aqueous solutions of Pd NCs, ligands (methionine), metal precursors (NH_4_)_2_PdCl_4_, and reducing agent (ascorbic acid). The synthesized Pd NCs exhibited a clear absorption band centered at 400 nm. In contrast, pure methionine, (NH_4_)_2_PdCl_4_, and ascorbic acid did not show any notable absorption.

**Figure 3:**
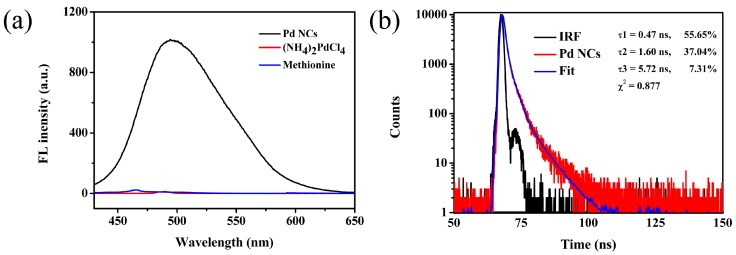
(**a**) Fluorescence spectra of prepared Pd NCs, (NH_4_)_2_PdCl_4_ and methionine solution; (**b**) Fluorescence lifetimes of Pd NCs in aqueous solution.

Moreover, neither (NH_4_)_2_PdCl_4_ nor pure methionine emit any fluorescence under the same conditions, the luminescence can be attributed to the synthesized Pd NCs (Figure 3a). Hence, the prepared Pd NCs featured strong photoluminescence property. The photoluminescence lifetime is also an important feature of fluorescent materials. Figure 3b shows a fitting curve of the luminescence decay of Pd NCs. The decay curve could be fitted by a three-exponential function with lifetime components at 0.47 ns (55.65%), 1.59 ns (37.04%), and 5.72 ns (7.31%). The calculated average fluorescence lifetime of the palladium clusters was 1.27 ns. Luminescent metal clusters often show markedly different luminescence lifetimes from those of bulk materials. Nano-second lifetimes, can be mainly attributed to the transition of electrons between different discrete levels in the metal cluster nuclei [36,37].

On the basis of the results of the Stokes shift and average luminescence lifetime, the fluorescence of the Pd NCs is suggested to belong to the band gap transition of a single state. The source of luminescence is the metal nucleus of the metal cluster, and the luminescent lifetime of the prepared palladium cluster is of the order of nanoseconds which indicates fluorescence. The fluorescence quantum yield of the nanoclusters was ~5.47% (calculated relative to Rhodamine 101), which is comparable to the yields reported for other metal nanoclusters stabilized by amino acids and higher than those reported for methionine-stabilized fluorescent Au NCs [38].

**Figure 4:**
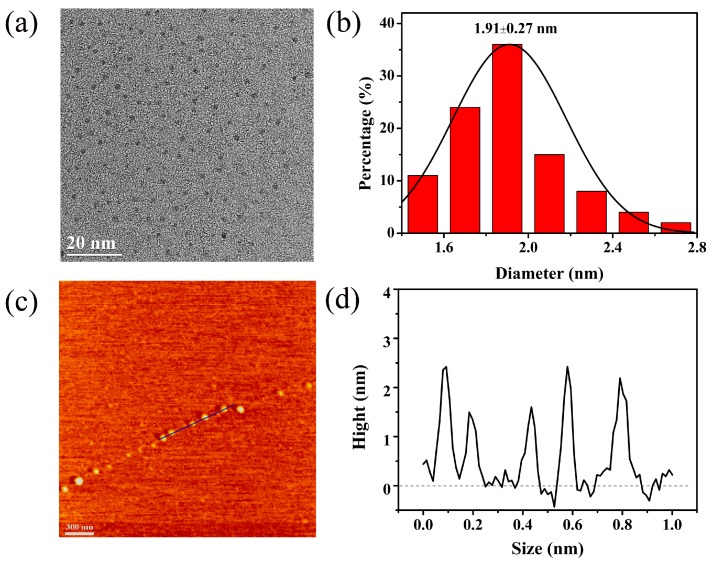
A typical HRTEM image (**a**) and particle size distribution histogram (**b**) of Pd NCs; AFM image of the Pd NCs (**c**) and its corresponding height profile (**d**).

To further characterize the morphology and the size distribution of the Pd NCs, the synthesized Pd NCs were characterized by high-resolution transmission electron microscopy (HRTEM) and atomic force microscopy (AFM). The results of HRTEM (Figure 4a) showed that, after protection by methionine and reduction by ascorbic acid, the particle size was uniform, with irregular spherical particles, showing a narrow size distribution. Particle counting and statistical analysis (Figure 4b) indicated that the synthesized palladium nanoclusters showed a relatively narrow size distribution in the range of 1.5–2.7 nm with a mean diameter of approximately 1.91 nm. The thickness of the Pd NCs was further characterized by AFM (Figure 4c). The typical topographic height was less than 2.5 nm (Figure 4d), indicating that we successfully prepared small fluorescent palladium nanoclusters.

### 3.3. Stability of Fluorescent Pd NCs

**Figure 5:**
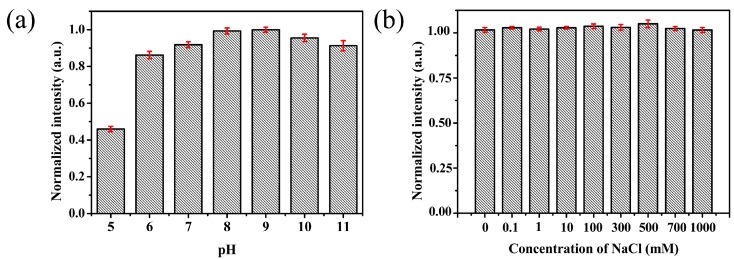
Fluorescence stability of Pd NCs under various pH values (**a**) and different concentration of NaCl (**b**).

The environmental stability of fluorescent nanoclusters is an important factor for practical applications. The effects of pH and ionic strength on the stability of the NCs were investigated. As shown in Figure 5a, the Pd NCs showed stable fluorescence over a pH range of 6 to 8, and weak fluorescence at pH = 5. This result can be attributed to the reduction of methionine, which is less stable in acidic environments owing to demethylation. The effects of ionic strength were examined in NaCl solution (0.1–1000 mM). Figure 5b shows that the fluorescence intensity of the Pd NCs was unaffected by a high ionic strength. 

### 3.4. Fluorescent Sensing of Hemolobin Using Pd NCs

**Figure 6:**
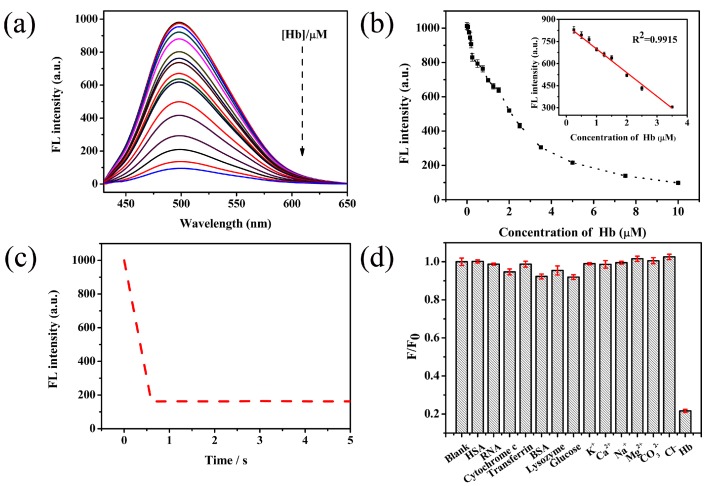
(**a**) Fluorescence response of the Pd NCs in the presence of various concentration of hemoglobin (0.05, 0.1, 0.15, 0.2, 0.25, 0.5, 0.75, 1, 1.25, 1.5, 2, 2.5, 3.5, 5, 7.5, and 10 µM); (**b**) Plots showing relationship between fluorescence intensity and hemoglobin concentration. Inset shows the hemoglobin linear detection range of 0.25–3.5 µM; (**c**) Response time of Pd NCs introducing 5 µM hemoglobin; (**d**) Selective experiments of Pd NCs for competitive molecules (5 µM).

Hemoglobin (Hb) is a protein presenting in higher organisms, which is responsible for carrying oxygen [39,40]. It transports oxygen from the lungs to every part of body through the blood stream. The content of hemoglobin in blood is closely related to many diseases—such as leukemia, anemia, and heart disease—such that its expression level is closely linked with biological functions [41,42]. In this study, we found that when hemoglobin solution was added to the Pd NCs solution, the fluorescence intensity of the Pd NCs gradually decreased as the hemoglobin concentration was increased (Figure 6a). When the hemoglobin concentration was in the range of 0.25–3.5 µM, a linear response emerged, with a correlation coefficient R^2^ of 0.9915 (Figure 6b). The detection limit was 50 nM. These data indicate that our Pd NCs have the ability to detect hemoglobin over a wide and low concentration range. Notably, the response time of the Pd NCs to hemoglobin was rapid. Within approximately 0.6 s, the quenching of the Pd NCs by hemoglobin reached equilibrium. These findings demonstrate the effectiveness of our established method for hemoglobin detection by Pd NCs (Figure 6c). Furthermore, we explored the effects of interfering substances on detection of hemoglobin to further determine the selectivity of Pd NCs. HSA, RNA, cytochrome C, transferrin, BSA, lysozyme, glucose, K^+^, Ca^2+^, Na^+^, Mg^2+^, CO_3_^2−^, and Cl^−^ were added to the Pd NCs solution to observe interference effects. These test results showed that the Pd NCs maintained their clear response to hemoglobin, suggesting that the Pd NCs have good selectivity for hemoglobin detection (Figure 6d).

## 4. Conclusions

In summary, we present a simple, non-toxic, harmless, environmentally friendly method for the synthesis of fluorescent Pd NCs. The method uses amino acids with a high biocompatibility as protective agents and (NH_4_)_2_PdCl_4_, which has good water solubility, as a metal precursor. The prepared Pd NCs were well dispersed in water with a uniform particle size distribution, strong blue-green fluorescence, and good biocompatibility. We found that the synthesized Pd NCs were sensitive to hemoglobin and could be successfully applied to detection of hemoglobin. Owing to the inherently excellent biocompatibility and detection performance, our fluorescent Pd NCs show promise for various applications in biosensing.
